# Rapid diagnosis of *Plasmodium falciparum* malaria using a point-of-care loop-mediated isothermal amplification device

**DOI:** 10.3389/fcimb.2022.961832

**Published:** 2022-08-19

**Authors:** Madhu Puri, Harsimran Kaur Brar, Evanka Madan, Rajesh Srinivasan, Kapil Rawat, Sai Siva Gorthi, Geeta Kumari, Raj Sah, Sashi Bhusan Ojha, Subhendu Panigrahi, Gunanidhi Dhangadamajhi, Rohini Muthuswami, Shailja Singh, Rentala Madhubala

**Affiliations:** ^1^ School of Life Sciences, Jawaharlal Nehru University, New Delhi, India; ^2^ Department of Instrumentation and Applied Physics, Indian Institute of Science, Bengaluru, India; ^3^ Special Centre for Molecular Medicine, Jawaharlal Nehru University, New Delhi, India; ^4^ Department of Biotechnology, Maharaja Sriram Chandra Bhanjadeo University, Baripada, India; ^5^ Department of General Medicine, VSS Medical College and Hospital, Sambalpur, India

**Keywords:** LAMP, *plasmodium falciparum*, diagnosis, LOD, parasite density, ADC value

## Abstract

LAMP diagnosis of malaria is simple and cost-effective with acceptable sensitivity and specificity as compared to standard diagnostic modules such as microscopy, RDTs and nested PCR, and thus its deployment for onsite screening of malaria in resource-limited regions is under consideration. However, the requirement of an electricity-operated dry bath and bulky read-out unit is still a major concern. In an effort to simplify this limitation, we have developed a portable LAMP device and fluorescence readout unit which can be used in the rapid point-of-care diagnosis of malaria. We have developed a point-of-care diagnostic LAMP device that is easy to operate by a mobile application, and the results can be quantified with a fluorescent readout unit. The diagnostic performance of the device was evaluated in 90 P*. falciparum*-infected clinical isolates stored at 4°C for 6-7 years and 10 freshly collected isolates from healthy volunteers. The LOD and quantitative ability of LAMP in estimating parasitemia levels were revealed with laboratory-grown *P. falciparum* strain (3D7). The LAMP assay performed in our device was exclusive for *P. falciparum* detection with sensitivity and specificity determined to be 98.89% and 100%, respectively, in clinical isolates. The LOD was documented to be 1 parasite/µl at the cut-off ADC value of 20. Parasite density estimated from ADC values showed concordance with microscopically determined parasite density of the cultured *P. falciparum* 3D7 strain. The LAMP assay performed in our device provides a possible portable platform for its deployment in the point-of-care diagnosis of malaria. Further validation of the quantitative ability of the assay with freshly collected or properly stored clinical samples of known parasitemia is necessary for field applicability.

## Introduction

Malaria is a global health problem with an estimated 241 million cases and 627,000 deaths reported in 2020 (WHO, 2021). Human malaria is caused by six species of *Plasmodium* (*P. falciparum, P. vivax, P. malariae, P. ovale, P. knowlesi* and *P. simium*). Early diagnosis with prompt and species-specific effective treatment plays a crucial role in the improved care of patients and subsequent outcomes. Because clinical diagnosis is non-specific and unreliable, the World Health Organization (WHO) recommends treatment of only parasitologically confirmed cases of malaria with appropriate anti-malarial drugs. While microscopy serves as the gold standard for malaria diagnosis, detection of parasite-specific antigens/antibodies by rapid diagnostic tests (RDTs) has been a widely adopted point-of-care diagnosis method (Kotepui et al., 2020). However, malaria diagnosis by both these methods is challenging when the parasite density is low or the patient has been recently treated. While the limit of detection (LOD) by microscopy is about 88 parasites/µl of blood (Joanny et al., 2014), it is about 100-200 parasites/µl of blood by RDT (Mwesigwa et al., 2019). Interestingly, almost all the RDTs use histidine-rich protein-2 (HRP2) antigen for *P. falciparum* detection because of its exclusive expression in all stages of *P. falciparum* growth in the blood (Rock et al., 1987). However, recent studies have reported reduced sensitivity of HRP2 based RDTs in different malaria-endemic regions, especially in regions where the circulating populations of *P. falciparum* have *pfhrp2* gene deletion or there is genetic variability in the target epitopes (leading to its presence or absence and copy number variation) within PfHRP2 (Poti et al., 2020).

Although a significant reduction in malaria incidence and deaths has been achieved over the last few decades, failure to detect asymptomatic patients and sub-microscopic infections in surveillance studies contributes to ongoing transmission and is a major threat to malaria control and elimination goals. In light of this, nucleic acid-based detection is promising because of its high sensitivity and specificity. The most reliable among these is diagnosis by nested PCR which has a LOD of 1 to 0.1 parasites/µl of blood (Wang et al., 2014). However, the requirement of expensive equipment and reagents and long turnaround time (due to sample processing to remove inhibitors of polymerase from the body fluid, reaction time and post-PCR events to visualize the amplified products) render PCR-based methods to be inadequate for point-of-care diagnosis and routine diagnosis in resource-limited settings.

Loop-mediated isothermal amplification (LAMP) method of malaria diagnosis is emerging as a recent alternative to PCR and can be employed directly on whole blood samples (with minimal processing) and purified DNA or RNA. Further, LAMP diagnosis of malaria has excellent sensitivity (as low as 0.5 to 0.05 parasites/µl) comparable to that of PCR and turnaround time similar to RDTs depending upon the target DNA, improvement of read-out procedures and mitigation of DNA extraction process (Sattabongkot et al., 2014). However, commercial LAMP kits often require a bulky dry bath and a read-out unit such as a fluorescence spectrophotometer which restricts the applicability of LAMP in fieldwork or point-of-care analysis. Therefore, we have developed a portable, rapid, point-of-care LAMP assay system that comprises a LAMP device and a fluorescence readout unit for the diagnosis of *P. falciparum* malaria. Further, although LAMP is shown to be promising in diagnosing asymptomatic patients and sub-microscopic infections (Cook et al., 2015; Katrak et al., 2017), whether it is comparable to PCR in detecting malaria infection in stored and degraded blood samples is still unknown. Until now, LAMP has been used for the qualitative detection of malaria infection, however, its quantitative potential for estimating parasitemia level has been largely overlooked. Therefore, in the present study, we aimed to compare the diagnostic performance of the LAMP assay performed in our device with PCR using stored and degraded blood samples which were previously confirmed for *P. falciparum* infections by microscopy, RDT and PCR. Moreover, the ability of the LAMP assay in estimating sample parasitemia has also been demonstrated using the laboratory-grown *P. falciparum* strain (3D7).

## Materials and methods

### Chemicals

Bst DNA polymerase large fragment, magnesium sulphate and deoxynucleotide phosphates (dNTPs) were purchased from NEB (Ipswich, MA, USA). Thermo Pol Buffer was provided with the enzyme. Betaine, hypoxanthine and sodium bicarbonate were brought from Sigma-Aldrich (St. Louis, MO, USA). AlbuMax I was purchased from Gibco, Grand Island, NY, USA, and SYBR Gold nucleic acid stain (10,000x concentration) was purchased from Thermo Fischer Scientific (Waltham, MA, USA). RPMI 1640 media was obtained from Invitrogen, Carlsbad, CA, USA. All other materials used were of analytical grade and commercially available.

### Parasite culture and isolation of dna

*P. falciparum* 3D7 strain was cultured in O+ve human erythrocytes (obtained from Rotary blood bank, New Delhi, India) at 2% hematocrit and incubated with mixed gas (5% O2, 5% CO_2_ and 90% N_2_) condition in RPMI 1640 media supplemented with hypoxanthine (27.2 mg/L), sodium bicarbonate (2 gm/L) and AlbuMax I (0.5 gm/L) based on an established protocol (Trager and Jensen, 1976). Cultures of 3D7 maintained at 10% parasitemia were used for *P. falciparum* DNA isolation using the phenol-chloroform extraction method.

### Study population and DNA isolation from clinical samples

Blood samples used for the detection of *P. falciparum* infections in LAMP assay were collected from Sambalpur, Odisha, India during 2012-2014, the details of which including inclusion and exclusion criteria are mentioned in our previous publication Dhangadamajhi et al., 2019. About 90 of these randomly selected samples stored at 4°C for about 6-8 years, positive for *P. falciparum* infections in the peripheral blood smear and confirmed by the species-specific PCR diagnosis method of Snounou et al. (1993), were used for DNA isolation by the phenol-chloroform method as described previously (Panigrahi et al., 2016). RDT test (SD Bioline) of about 40 of these samples also confirmed *P. falciparum* infection. Freshly isolated DNA from 10 healthy volunteers was used as a negative control.

### LAMP primer design

The sequences for 6 set of primers to amplify *P. falciparum* 18SrRNA were taken from (Han et al., 2007). Primer sequences are given in **Table 1**.

**Table 1:** List of LAMP primers used.

Primer	Sequence
Forward inner primer (FIP)	5’ AGCTGGAATTACCGCGGCTG GGTTCCTAGAGAAACAATTGG 3’
Backward inner primer (BIP)	5’ TGTTGCAGTTAAAACGTTCGTAGCCCAAACCAGTTTAAATGAAAC 3’
F3	5’ TGTAATTGGAATGATAGGAATTTA 3’
B3c	5’ GAAAACCTTATTTTGAACAAAGC 3’
LPF	5’ GCACCAGACTTGCCCT 3’
LPB	5’ TTGAATATTAAAGAA 3’

### LAMP assay

LAMP assay was performed in the device as previously described (Puri et al., 2021). Briefly, a working dilution of 10X was prepared from primer stocks (100 µM). This 10 X dilution comprised of 16 µM FIP, 16 µM BIP, 8 µM LPF, 8 µM LPB, 2 µM F3 and 2 µM B3C primers. The LAMP reaction contained 1X primer mix, 1X Thermo Pol buffer, 1.4 mM dNTPs, 6 mM MgSO4, 8 units of Bst DNA polymerase, 0.8 M betaine and template DNA. Nuclease-free water was added to the reaction to make up the volume to 25 µl. To check the lowest concentration at which LAMP assay can detect infection, a reaction was set up with serial dilutions containing 0.000001%, 0.00001%, 0.00005%, 0.0001%, 0.0005%, 0.001%, 0.01%, 0.1%, 1% and 10% parasitemia of *P. falciparum* 3D7 genomic DNA. As a control, DNA from a sample with zero parasitemia, i.e., only blood, was also subjected to LAMP assay. Clinical samples were diluted to 100 pg to use as templates in the LAMP reaction. A non-template control was used as a negative control in every reaction. To ascertain that the LAMP primers were specific to *P. falciparum*, they were tested with genomic DNA from *P. vivax* and *L. donovani*. The reaction was performed at 60°C for 100 min in the LAMP device.

### Detection of LAMP amplification

Two µl of SYBR gold dye was added to the reaction mixture to detect the amplification. Before use, the dye was diluted 10 times in nuclease-free water. SYBR gold is color sensitive and in the presence of amplification, it changes its color from orange to green. The change was visible with the naked eye and for quantification, LAMP products were placed inside the readout unit which measured the fluorescence intensity upon amplification (ADC values). The ADC values were directly proportional to the amount of amplified product. Amplification was also confirmed by electrophoresing 3 µl of the reaction mixture on a 2.5% agarose gel followed by staining it with ethidium bromide for visualization. LAMP positive samples displayed the characteristic ladder-like pattern.

### LAMP amplification device and fluorescence readout unit

**Figure 1:**
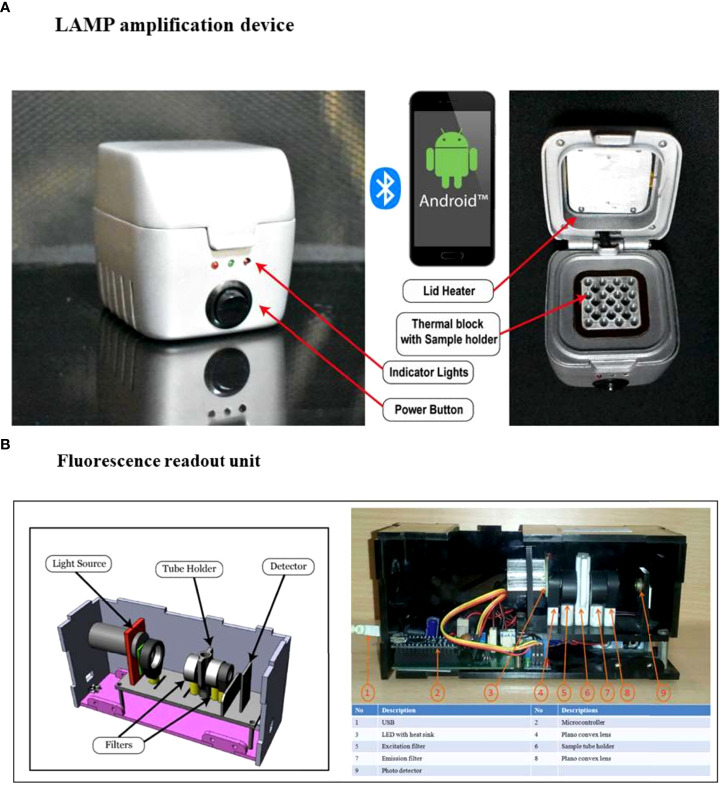
Components of the portable LAMP device and fluorescence readout unit.**(A)** The LAMP device has 16 sample wells, a heating thermal block and a lid which features a heating element to prevent condensation during the amplification process. Three indicator lights in the front panel: red to show the operation status of system power, orange to show the Bluetooth connection, and green to display the amplification reaction process, are present. The reaction settings are set using a mobile application that connects the LAMP device to a smartphone. The app has a simple graphical user interface to set the temperature and time for the reaction and shows the real-time temperature of the block. **(B)** The fluorescence readout unit has an integrated LED light source, optical filters (excitation and emission filters suitable for SYBR Gold), a sample vial holder, a photodiode, and an electronic driving circuit. The samples are mixed with SYBR Gold and placed in the sample holder. When the measurement is initiated the LED and photodiode are synchronized to read the fluorescence emission from the sample. The system measures the fluorescence levels at an interval of 3 seconds (1 second on and 2 seconds off). The data is logged into a file where it can be used for further analysis (Puri et al., 2021).

The LAMP amplification device which can be operated *via* a mobile application and fluorescent readout unit used in this study were the same as described by Puri et al., 2021. Briefly, the core of the LAMP device consists of a resistive heating block and a system controller placed in a 3D printed casing shown in (**Figure 1A**). The heating block heats and holds samples at specific temperatures, ranging between 40–90°C, and the system controller maintains the specific temperature and time duration set by the user. The reaction temperature for amplification can be set between 50-72°C. For point-of-care applications, it is imperative to provide the test results immediately after completion of the test, therefore, a portable LAMP readout unit has been developed (**Figure 1B**). This unit is designed to analyze the test samples using nucleic acid intercalating dyes, such as the SYBR Gold nucleic acid stain. The components include a high-power LED with a wider spectrum (LXML-PE01-0070-High Power LEDs - Single Color Cyan 70lm, 350mA wavelength range of 490-515nm), an excitation filter between the LED and sample (CWL 490nm, Dia 12.5mm, BW: 15nm for excitation), a 15 mm focus lens, an emission filter (CWL 520 nm, Dia12.5 mm, BW: 15 nm), a collector lens, and an LTC 1051 op-amp. The signal from the op-amp is read by the ADC pin of the microcontroller (Atmega328P) and can be recorded on a laptop using the software. The emitted fluorescence values are read at a 3-second interval wherein the LED is on for 1 second and off for the remaining 2 seconds. Our LAMP system has an edge over other LAMP systems in the market, as it can test a higher number of samples (16 samples) simultaneously than the Axxin Molecular T8 and Diagenetix Bioranger systems (8 samples) and the Dialunox ESEQuant TS2 (12 samples), and is more compact than the Agdia AmplifyRP system.

### Statistical analysis

LAMP assay ADC value data were analyzed by GraphPad prism and represented as mean ± standard error of the mean (S.E.M.). A standard curve was plotted using log-transformed parasitemia value on the x-axis and ADC value on the y-axis. For the prediction of parasitemia from the ADC value, the inverse logarithm value was calculated by raising the base10 to the logarithm of the log-transformed parasitemia value corresponding to a different ADC value. The percentage sensitivity, specificity and accuracy with 95% confidence intervals (CI) were calculated using Medcalc software as previously described by Puri et al., 2021.

## Results

### Exclusivity of the LAMP assay performed in the device

**Figure 2:**
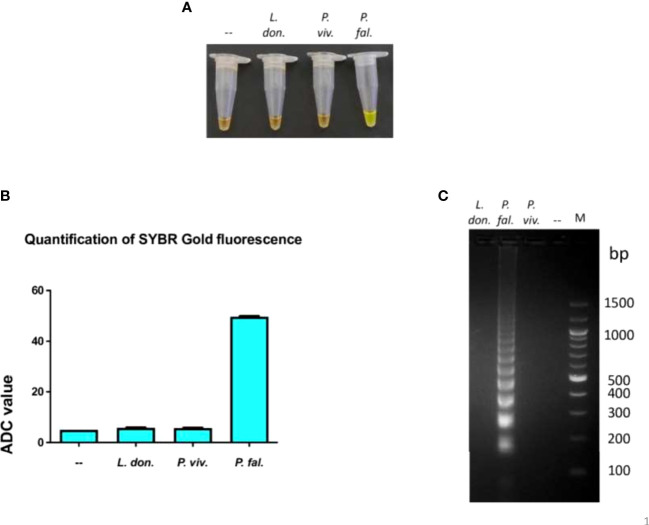
Exclusivity of the LAMP reaction performed in the device.The exclusivity of the LAMP reaction performed in the device was determined. One ng of genomic DNA from each of *L. donovani* Bob, *P. vivax* and *P. falciparum* strains was used in a LAMP reaction along with *P. falciparum* LAMP primers for 100 minutes, as described in Materials and Methods. **(A)** The confirmation of LAMP amplification was done by SYBR Gold detection. Diluted SYBR Gold nucleic acid stain was added to the LAMP products, and the color change was detected by visual examination. –: non-template control. **(B)** The quantification of SYBR Gold fluorescence was done by measuring the fluorescence intensity of the samples by a detection device, designated as ADC values. The mean + SEM of 10 ADC values are plotted for each sample. **(C)** Electrophoresis of LAMP products on a 2.5% agarose gel. M: 100 bp DNA ladder.

To determine if the LAMP assay performed in the device was exclusive to *P. falciparum*, 1 ng of genomic DNA from *Leishmania donovani* Bob strain and *P. vivax* was subjected to LAMP assay in the device using primers specific to *P. falciparum* 18S rRNA. One ng of genomic DNA from *P. falciparum* was taken as a positive control in the same assay. To visualize amplification, one aliquot of LAMP products was incubated with SYBR Gold stain and another aliquot was electrophoresed on an agarose gel. There orange to green color change with SYBR Gold addition and the characteristic ladder-like band pattern was observed only in the *P. falciparum* sample and not in the *L. donovani* and *P. vivax* samples, thereby indicating that the 18S rRNA primers did not amplify *L. donovani* and *P.vivax* DNA and were specific to *P. falciparum* (**Figures 2A–C**). Therefore, these experiments demonstrate that the LAMP assay performed in the device was exclusive to *P. falciparum*.

### Quantification ability and limit of detection of *P. falciparum* DNA amplified in the LAMP device

**Figure 3:**
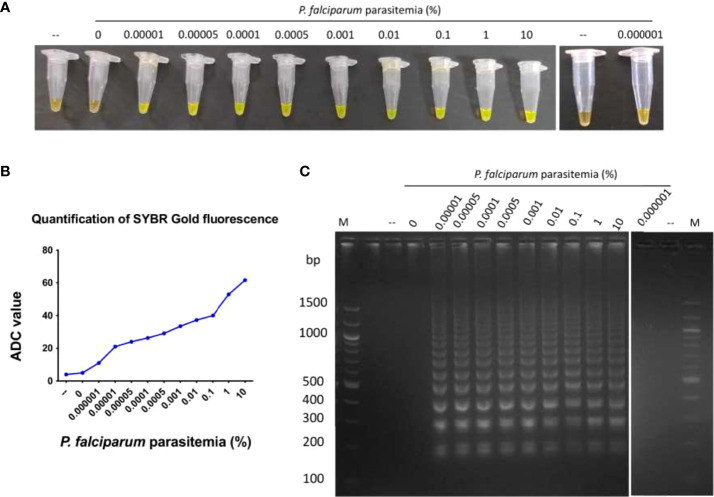
Limit of detection of *P. falciparum* genomic DNA amplified in the LAMP device.DNA isolated from *P. falciparum* cultures containing 0.000001 to 10% parasitemia was used in a LAMP reaction for 100 minutes, as described in Materials and Methods. As a control, DNA isolated from a sample containing zero parasitemia, i.e. only blood, was also tested in the LAMP reaction. **(A)** The confirmation of LAMP amplification was done by SYBR Gold detection. Diluted SYBR Gold nucleic acid stain was added to the LAMP products, and the color change was detected by visual examination. –: non-template control. **(B)** The quantification of SYBR Gold fluorescence was done by measuring the fluorescence intensity of the samples by a detection device, designated as ADC values. The mean + SEM of 10 ADC values are plotted for each sample. **(C)** Electrophoresis of LAMP products on a 2.5% agarose gel. M: 100 bp DNA ladder.

An essential parameter to evaluate the analytical performance of the LAMP device was to calculate the limit of detection of *P. falciparum* DNA that can be amplified. For this, DNA isolated from *P. falciparum* cultures with 10% parasitemia serially diluted to 0.000001% parasitemia was tested for amplification. A negative control sample with zero parasitemia, i.e., only blood, was also included in the reaction. It was observed that DNA from cultures containing 0.00001 to 10% parasitemia could be amplified in the LAMP device, and the zero parasitemia control sample was not amplified (**Figures 3A–C**). Therefore, the limit of detection of the LAMP device was 1 parasite/µl of *P. falciparum* culture (as estimated by counting the number of trophozoites/100 RBCs). To ascertain if parasitemia can also be estimated from ADC value, 1 ng of genomic DNA isolated from different parasitemia of *P. falciparum* cultures (0.5, 1, 2 and 5%) were used in the LAMP assay. The ADC value obtained against these samples was used for parasite density estimation using the calibration equation (y = 6.679x+52.13, R2 = 0.9751) of serially diluted genomic DNA of *P. falciparum* (**Figure 3B**). The results showed an excellent agreement of parasitemia estimated from ADC value with parasitemia value determined by microscopy (**Table 2**).

**Table 2:** Comparison of parasitemia estimated from ADC value with parasitemia determined by microscopy.

Parasitemia (%)	ADC value	Parasitemia estimated from ADC value
0.5	50	0.479
1	52	0.954
1.5	53	1.347
2	54	1.902
5	57	5.35

### Detection of *P. falciparum* DNA in patient samples

**Figure 4:**
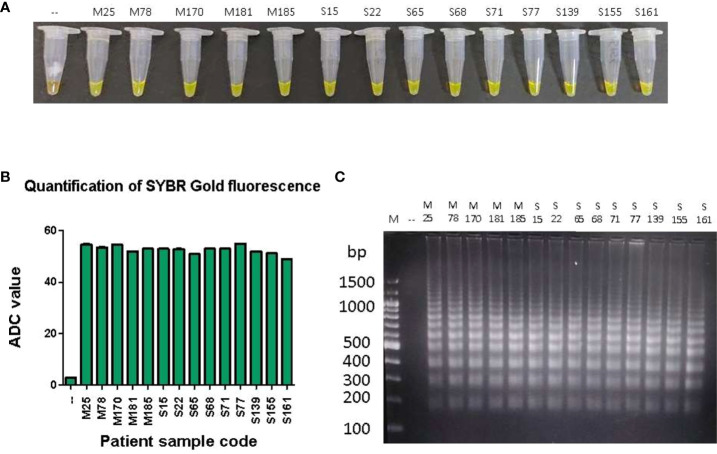
LAMP amplification of *P. falciparum* DNA from patient samples.One hundred pg of DNA from 90 patient samples were used in a LAMP reaction for 100 minutes, as described in Materials and Methods. **(A)** The confirmation of LAMP amplification was done by SYBR Gold detection. Diluted SYBR Gold nucleic acid stain was added to the LAMP products, and the color change was detected by visual examination. –: non-template control. **(B)** The quantification of SYBR Gold fluorescence was done by measuring the fluorescence intensity of the samples by a detection device, designated as ADC values. The mean + SEM of 10 ADC values are plotted for each sample. **(C)** Electrophoresis of LAMP products on a 2.5% agarose gel. M: 100 bp DNA ladder.

To test the ability of the LAMP device to detect and amplify *P. falciparum* DNA in clinical samples, DNA isolated from the stored blood of 90 malaria patient samples (which were previously confirmed for *P. falciparum* infections by standard diagnostic tests such as Quantitative Buffy Coat (QBC) and PCR were used in a LAMP assay in our device (**Table S1**). Additionally, 40 of these samples were also randomly tested positive by RDT (**Table S1**). In the LAMP assay, 89 samples were found to be positive and 1 was negative (**Table S1**). A representative image of LAMP assay data from 10 clinical samples is shown in (**Figures 4A–C**). In clinical samples, we did not find any correlation between ADC values and parasitemia. As a control, freshly isolated DNA from 10 healthy volunteers was also tested in the LAMP assay in our device and no amplification was observed (**Supplementary Figures 1A–C**).

### Comparison of diagnostic test evaluation parameters of QBC, ICT and *P. falciparum* PCR tests with the LAMP assay

An evaluation of the analytical performance of the LAMP assay, in comparison to that of QBC, ICT and *P. falciparum* PCR tests was performed (**Table 3**). Out of the total 100 (90 positive and 10 healthy volunteer) samples tested in the LAMP device, 1 false-negative and no false-positive results were obtained. The LAMP assay performed in the device demonstrated excellent sensitivity (98.89%; 95% CI= 93.96-99.97), specificity (100%; 95% CI= 69.15% to 100.00%) and accuracy (99.01%; 95% CI= 94.61% to 99.97%).

**Table 3:** Comparison of diagnostic test evaluation parameters of ITS-1 PCR and LAMP assay.

Parameters	QBC	RDT	PCR	LAMP assay
True positives	90	40	90	89
False negatives	0	0	0	1
True negatives	10	10	10	10
False positives	0	0	0	0
Sensitivity (%) (95% CI)	100.00 (95.98 - 100.00)	100.00 (91.19 - 100.00)	100.00 (95.98 - 100.00)	98.9 (94.03 - 99.97)
Specificity (%) (95% CI)	100 (69.15 - 100.00)	100 (69.15 - 100.00)	100 (69.15 - 100.00)	100 (69.15 - 100.00)
Accuracy (%) (95% CI)	100 (96.38 - 100.00)	100 (92.89 - 100.00)	100 (96.38 - 100.00)	99.01 (94.61 - 99.97)

Confidence intervals for sensitivity are exact Clopper-Pearson confidence intervals. Confidence intervals for predictive values are the standard logit confidence intervals given by (Mercaldo et al., 2007).

## Discussion

The LAMP-based detection of human malaria exhibits acceptable sensitivity and specificity as compared to microscopy, RDTs and nested PCR, irrespective of *Plasmodium* species (Han et al., 2007; Paris et al., 2007; Yamamura et al., 2009). Further, LAMP being less expensive, simple and having the potential of amplifying both RNA and DNA at a constant temperature, its deployment for molecular testing of malaria in resource-limited regions is under consideration. However, the bulkiness of the dry bath and readout unit is still a major concern that may be likely to restrict the field applicability of LAMP in point-of-care diagnosis. In an effort to simplify this limitation, we have developed a compact LAMP device and fluorescence readout unit, both of which are portable, and have the potential to be used in the rapid point-of-care diagnosis of malaria.

Recently, considerable progress has been made in the development of LAMP diagnostic systems for malaria diagnosis. A LAMP-based lateral flow device method has been devised for the detection of malaria that uses biotin-labeled and fluorescein amidite-labeled loop primers in the LAMP reaction, and the end-product can be visualized on a dipstick (Mallepaddi et al., 2018). This study tested 90 samples of *P. knowlesi* infection, 49 samples of *P. falciparum* infection, 56 samples of *P. vivax* infection, 15 samples of mixed infection with *P. falciparum* and *P. vivax*, 60 non-malaria infected human samples, one of *Toxoplasma gondii* infection, one of *Sarcocystis* spp. infection, and eight healthy donor samples. Their results indicated that all 90 P*. knowlesi* and *P. vivax* samples were positively amplified by the LAMP-LFD assay. However, one *P. falciparum* and one mixed-infection sample was not amplified, possibly because of low parasitemia or DNA degradation. Another study has reported the development of a paper-based microfluidic device that enables multiplex LAMP-based detection of malaria in the blood (Reboud et al., 2019). Although this offers a very cheap and simple alternative to the conventional LAMP assay, however, local climatic conditions like variations in temperature and humidity can affect test results by altering the rates of evaporation and speed of fluid movement during sample introduction into microfluidic matrices. A commercial Loopamp MALARIA Pan Detection Kit has also been developed by Eiken Chemical Co., Ltd., Tokyo, Japan, with a LOD of 20 parasites/µl for *P. falciparum* (Piera et al., 2017), which is considerably higher than that obtained in the LAMP assay performed in our device (1 parasite/µl).

Our LAMP system offers numerous advantages over contemporary LAMP diagnostic modules. The LAMP device is Bluetooth-enabled, compact, light-weight, and portable. It is also very user-friendly, as it is operated and controlled *via* a dedicated mobile application, thereby making remote operation possible. The fluorescent readout unit can be connected to a computer/laptop for recording ADC values and subsequent data analysis. More importantly, because assay cost is a limiting factor for diagnosis in under-developed and developing countries where malaria is endemic, we have developed a cost-effective diagnostic testing system in which one test costs less than 1.5 USD.

Using *P. falciparum*-specific LAMP primers (Han et al., 2007), we successfully diagnosed *P. falciparum* infections in previously confirmed stored blood samples with 98.89% sensitivity and 100% specificity by our LAMP device. The LAMP assay could not detect *P. falciparum* infection in one sample. The same sample could also not be amplified with *P. falciparum*-specific primers in a nested PCR test, which indicates a possible degradation of parasite DNA. Interestingly, no color change was detected in the samples containing genomic DNA for *P. vivax, L. donovani* and healthy human volunteers, which depicts the exclusivity of the assay for *P. falciparum* DNA. In the present study, the LOD was determined to be as low as 0.00001% parasitemia (which is about 1 parasite/µl). Previous studies using similar LAMP primers have documented a LOD of 2 parasites/µl (Oriero et al., 2015). The LOD for parasite genomic DNA isolated by a simple heat-treated method is reported to be about 40 parasites/µl (Lucchi et al, 2016), whereas by chemical lysis with Illumigene lysis buffer is 0.5 parasites/µl (De Koninck et al, 2017). Further, LOD has been shown to vary with sample types (culture or clinical samples) and the base parasitemia level used for DNA isolation. Despite these variations, the excellent performance of our LAMP assay system could be due to the efficient extraction process for the high yield of parasite genomic DNA and the use of cultured parasites for DNA isolation. Since the present study aimed to validate the diagnostic performance of the LAMP assay system in diagnosing confirmed cases of *P. falciparum* infections, variations in LOD due to differences in DNA isolation processes, sample types, or base level parasite density were not examined. However, simple and rapid DNA isolation procedures like heat-treatment or rapid DNA extraction by chemical lysis seem to have field applicability in point-of-care LAMP diagnosis and require to be tested for determining the LOD.

Apart from the identification of *Plasmodium* species, the determination of parasite density is crucial for effective anti-malarial therapy and to understand the transmission dynamics and relative distribution of malaria species. However, LAMP-based diagnosis has been primarily used for qualitative screening. Microscopy and/or real-time PCR remain(s) essential for quantitative estimation of parasite density. Our approach to detecting *P. falciparum* infection through ADC value has an additional advantage of estimating parasite density. A cut-off ADC value of ~ 20 (value for 0.00001% parasitemia) represented the presence of *P. falciparum* infection. More specifically, a significant positive correlation between serially diluted parasite genomic DNA isolated from fixed parasitemia (10%) and their corresponding ADC values uncovered its potential for parasite density estimation. Based on the calibration equation of ADC value, using *in vitro* cultures of *P. falciparum*, we successfully estimated parasite density which corroborated with the parasitemia obtained by microscopy. However, our attempt of estimating parasite density could not be replicated with DNA isolated from stored blood samples. The failure to quantify parasite density in clinical samples could be due to the degradation of parasite DNA during storage, as stored samples that were previously *P. falciparum* PCR-positive when re-analyzed by LAMP assay and PCR were observed to be negative. Furthermore, almost all the LAMP-positive clinical samples had ADC values between 40 and 57 despite their major differences in parasite density at the time of sample collection (data not shown). This narrow range of ADC values and PCR-negative results of *P. falciparum*-infected stored blood samples ascertained the degradation of parasite DNA during storage. Therefore, the parasite density estimation potential of our LAMP-based assay needs to be validated with freshly collected and properly stored clinical samples for its possible application in the field.

Although LAMP diagnosis has been proven to be highly sensitive and specific in diagnosing symptomatic malaria infections, its performance against thawed and degraded blood samples still remains unknown. In light of this, the results of the present study demonstrating the superior diagnostic performance of our LAMP assay system with improperly stored blood samples highlights the diagnostic ability of LAMP in degraded samples. To our knowledge, this is the first study that has tested *P. falciparum* malaria infection by LAMP assay in stored blood samples with possible degradation of parasite genomic DNA. Besides symptomatic infections, LAMP is also proven beneficial in diagnosing asymptomatic malaria with low or sub-microscopic parasitemia (Cook et al., 2015; Katrak et al., 2017). Based on its performance with degraded samples, we anticipate similar or better efficacy of our LAMP assay system for low-density infections, however, for this, field studies with a larger number of samples are warranted. Further, our preliminary study conducted with P*. falciparum*-specific LAMP primers needs to be extrapolated with other human malaria-causing *Plasmodium* species in future studies. Our study is limited by the fact that the 90 PCR-positive *P.falciparum* clinical samples stored for about 6-8 years could not be re-tested for PCR positivity before their use in LAMP assay due to the non-availability of DNA in adequate amounts. Since the current PCR-positive status of these samples might not be the same as it was before 6-8 years due to sample age and storage conditions, our inclusion of PCR data performed 6-8 years ago may influence the observed sensitivity and specificity of our LAMP assay. However, consistent reports of false-negative results by both PCR and LAMP in one sample (due to possible degradation of DNA), and lack of false positivity by LAMP assay are the strengths of our LAMP test.

In conclusion, the reported LAMP assay system provides a possible portable platform for its deployment in the molecular testing of malaria in point-of-care diagnosis. Our validation of the quantitative potential of this system to estimate parasite density in laboratory-grown *P. falciparum* strain and its excellent diagnostic performance on stored and degraded clinical samples highlights its field applicability in the diagnosis of malaria. Future studies from our laboratory are directed towards testing freshly collected or properly stored symptomatic and asymptomatic clinical samples of known parasitemia using our LAMP device.

## Data availability statement

The original contributions presented in the study are included in the article/**Supplementary Material**. Further inquiries can be directed to the corresponding authors.

## Ethics statement

The studies involving human participants were reviewed and approved by IBSC, JNU, New Delhi (Ref Number: JNU/IBSC/2020/17. The patients/participants provided their written informed consent to participate in this study.

## Author contributions

Conceptualization, ReM, SG, SS and RoM; Methodology, ReM, SS, SG, GD, MP, RS and KR; Investigation, MP, HB, EM, GK, RSr, SO, RSa and SP; Writing- original draft, MP, HB, ReM, SS and GD; Writing-review and editing, MP, SS, GD, RoM, and ReM. Funding acquisition, ReM, RoM, SS, SG and GD; Supervision, ReM, RoM, SS, SG and GD. All authors contributed to the article and approved the submitted version.

## Funding

ReM was funded by EMR/2016/004948 from Science and Engineering Research Board, India (https://www.serbonline.in/SERB/HomePage do) and VI-D&P/569/2016-17/TDT/C from Department of Science and Technology, India (www.dst.gov.in). MP and EM were supported by the D. S. Kothari Post-Doctoral Fellowship from UGC. HB was supported by a fellowship from CSIR. The funders had no role in study design, data collection and analysis, decision to publish, or preparation of the manuscript.

## Acknowledgments

We thank the Central Instrumentation Facility at the School of Life Sciences, Jawaharlal Nehru University, for providing the instrumentation facility. ReM is an A. S. Paintal Distinguished Scientist Chair of ICMR. This research was partly supported by the Gore Subraya Bhat chair in Digital Health, awarded to SG, and Meity-DST.

## Conflict of interest

The authors declare that the research was conducted in the absence of any commercial or financial relationships that could be construed as a potential conflict of interest.

## Publisher’s note

All claims expressed in this article are solely those of the authors and do not necessarily represent those of their affiliated organizations, or those of the publisher, the editors and the reviewers. Any product that may be evaluated in this article, or claim that may be made by its manufacturer, is not guaranteed or endorsed by the publisher.
