# Supplementary figures and images for: Rapid diagnosis of Plasmodium falciparum malaria using a point-of-care loop-mediated isothermal amplification device
Source: Front Cell Infect Microbiol. 2022 Aug 19;12:961832. doi: 10.3389/fcimb.2022.961832 (PMC9437306; doi:10.3389/fcimb.2022.961832)

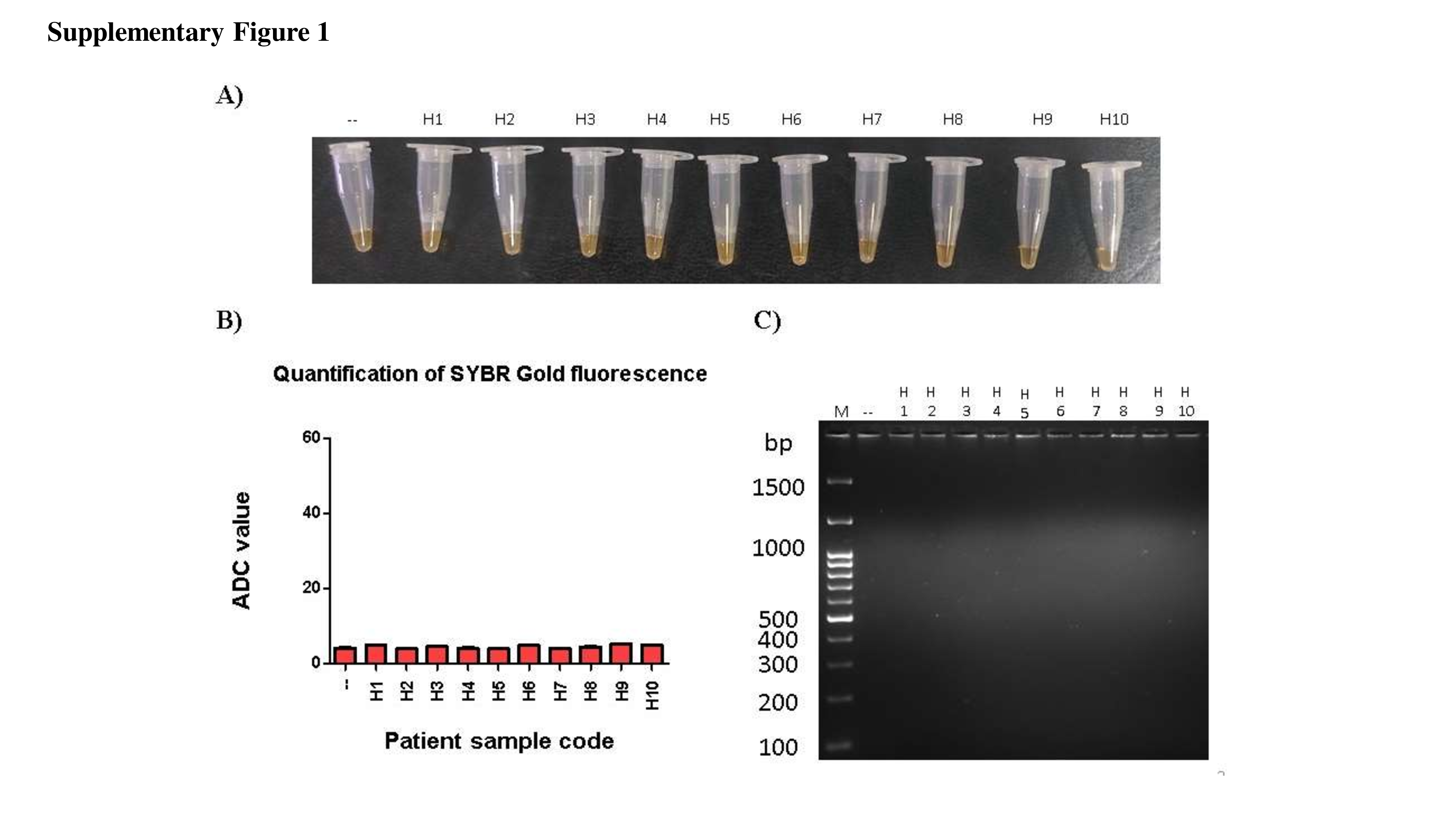

Supplement: Supplementary file 1 [file Image_1.tiff]
