# Supplementary material for: Rapid diagnosis of Plasmodium falciparum malaria using a point-of-care loop-mediated isothermal amplification device
Source: Front Cell Infect Microbiol. 2022 Aug 19;12:961832. doi: 10.3389/fcimb.2022.961832 (PMC9437306; doi:10.3389/fcimb.2022.961832)
Supplement: Supplementary file 2 [file Table_1.docx]

Supplementary Material

**Table S1. Comparison of QBC, ICT and *P. falciparum* PCR test results with those of LAMP assay in patient samples.**

| **S.No.** | **SAMPLE CODE** | **QBC**  **(fluorescence microscopy)** | **ICT**  **(RDT)** | **PCR**  ***P. f.* +ive** | **LAMP ASSAY RESULT** | **ADC value** |
| --- | --- | --- | --- | --- | --- | --- |
| 1 | S67 | +++ | Not tested | + | Positive | 55 |
| 2 | M149 | +++ | Not tested | + | Positive | 53 |
| 3 | M42 | ++ | Not tested | + | Positive | 57 |
| 4 | S115 | +++ | Not tested | + | Positive | 55 |
| 5 | S34 | ++ | Not tested | + | Positive | 54 |
| 6 | S108 | +++ | Not tested | + | Positive | 53 |
| 7 | S70 | ++ | Not tested | + | Positive | 55 |
| 8 | M182 | +++ | Not tested | + | Positive | 55 |
| 9 | S66 | ++++ | Not tested | + | Positive | 52 |
| 10 | M14 | + | Not tested | + | Positive | 57 |
| 11 | M152 | ++ | + | + | Positive | 56 |
| 12 | M11 | + | Not tested | + | Positive | 56 |
| 13 | M142 | +++ | Not tested | + | Positive | 56 |
| 14 | M176 | ++ | + | + | Positive | 51 |
| 15 | M73 | + | Not tested | + | Positive | 55 |
| 16 | M135 | ++ | + | + | Positive | 55 |
| 17 | M136 | + | Not tested | + | Positive | 55 |
| 18 | M141 | ++++ | + | + | Positive | 53 |
| 19 | M167 | + | + | + | Positive | 50 |
| 20 | M186 | + | Not tested | + | Positive | 54 |
| 21 | M190 | +++ | Not tested | + | Positive | 51 |
| 22 | S29 | ++ | + | + | Positive | 48 |
| 23 | S41 | ++++ | Not tested | + | Positive | 50 |
| 24 | S71 | + | + | + | Positive | 53 |
| 25 | S114 | ++ | + | + | Positive | 51 |
| 26 | S166 | ++++ | Not tested | + | Negative | 10 |
| 27 | S198 | ++ | Not tested | + | Positive | 54 |
| 28 | M25 | + | + | + | Positive | 55 |
| 29 | M78 | + | + | + | Positive | 54 |
| 30 | M170 | + | Not tested | + | Positive | 55 |
| 31 | M181 | + | Not tested | + | Positive | 52 |
| 32 | M185 | ++ | + | + | Positive | 53 |
| 33 | S15 | +++ | Not tested | + | Positive | 53 |
| 34 | S22 | ++ | Not tested | + | Positive | 53 |
| 35 | S65 | ++ | Not tested | + | Positive | 51 |
| 36 | S68 | ++ | + | + | Positive | 53 |
| 37 | S77 | + | + | + | Positive | 55 |
| 38 | S139 | + | + | + | Positive | 52 |
| 39 | S155 | +++ | Not tested | + | Positive | 51 |
| 40 | S161 | ++++ | Not tested | + | Positive | 49 |
| 41 | M34 | + | Not tested | + | Positive | 50 |
| 42 | M53 | + | Not tested | + | Positive | 52 |
| 43 | M132 | + | + | + | Positive | 49 |
| 44 | M172 | + | + | + | Positive | 53 |
| 45 | M184 | ++ | + | + | Positive | 51 |
| 46 | M199 | + | + | + | Positive | 49 |
| 47 | S20 | ++ | Not tested | + | Positive | 50 |
| 48 | S38 | +++ | Not tested | + | Positive | 51 |
| 49 | S81 | + | Not tested | + | Positive | 53 |
| 50 | S109 | + | + | + | Positive | 54 |
| 51 | S138 | +++ | Not tested | + | Positive | 49 |
| 52 | S147 | ++++ | Not tested | + | Positive | 53 |
| 53 | S168 | ++ | + | + | Positive | 52 |
| 54 | M72 | + | + | + | Positive | 46 |
| 55 | M147 | ++ | + | + | Positive | 32 |
| 56 | M150 | + | + | + | Positive | 49 |
| 57 | M157 | +++ | + | + | Positive | 50 |
| 58 | M161 | + | + | + | Positive | 55 |
| 59 | M162 | + | + | + | Positive | 47 |
| 60 | M196 | + | Not tested | + | Positive | 49 |
| 61 | S75 | ++++ | Not tested | + | Positive | 44 |
| 62 | S117 | + | Not tested | + | Positive | 46 |
| 63 | S132 | ++ | Not tested | + | Positive | 48 |
| 64 | S174 | + | + | + | Positive | 53 |
| 65 | S179 | ++ | Not tested | + | Positive | 44 |
| 66 | S200 | ++ | + | + | Positive | 28 |
| 67 | M138 | + | + | + | Positive | 54 |
| 68 | M151 | ++ | + | + | Positive | 52 |
| 69 | M159 | + | Not tested | + | Positive | 51 |
| 70 | M188 | +++ | + | + | Positive | 53 |
| 71 | S73 | +++ | Not tested | + | Positive | 53 |
| 72 | S134 | ++ | Not tested | + | Positive | 51 |
| 73 | S142 | ++ | + | + | Positive | 51 |
| 74 | S153 | ++ | Not tested | + | Positive | 55 |
| 75 | M67 | + | Not tested | + | Positive | 56 |
| 76 | M124 | ++ | + | + | Positive | 52 |
| 77 | M133 | +++ | Not tested | + | Positive | 43 |
| 78 | M160 | ++++ | Not tested | + | Positive | 51 |
| 79 | M192 | +++ | + | + | Positive | 55 |
| 80 | S83 | +++ | Not tested | + | Positive | 49 |
| 81 | S110 | ++ | Not tested | + | Positive | 49 |
| 82 | S74 | + | + | + | Positive | 48 |
| 83 | S120 | ++ | Not tested | + | Positive | 46 |
| 84 | S131 | ++++ | Not tested | + | Positive | 54 |
| 85 | S140 | ++ | Not tested | + | Positive | 51 |
| 86 | S172 | + | + | + | Positive | 44 |
| 87 | S178 | + | + | + | Positive | 49 |
| 88 | S78 | + | + | + | Positive | 42 |
| 89 | S24 | + | + | + | Positive | 51 |
| 90 | S167 | +++ | Not tested | + | Positive | 49 |
